# *Troglostrongylus brevior* and *Troglostrongylus subcrenatus* (Strongylida: Crenosomatidae) as agents of broncho-pulmonary infestation in domestic cats

**DOI:** 10.1186/1756-3305-5-178

**Published:** 2012-08-23

**Authors:** Emanuele Brianti, Gabriella Gaglio, Salvatore Giannetto, Giada Annoscia, Maria Stefania Latrofa, Filipe Dantas-Torres, Donato Traversa, Domenico Otranto

**Affiliations:** 1 Dipartimento di Sanità Pubblica Veterinaria, Facoltà di Medicina Veterinaria, Università degli Studi di Messina, Messina, Italy; 2 Dipartimento di Sanità Pubblica e Zootecnia, Facoltà di Medicina Veterinaria, Università degli Studi di Bari, Valenzano, BA, Italy; 3 Departamento de Imunologia, Centro de Pesquisas Aggeu Magalhães, Fundação Oswaldo Cruz, Recife, PE, Brazil; 4 Dipartimento di Scienze Biomediche Comparate, Facoltà di Medicina Veterinaria, Università degli Studi di Teramo, Teramo, Italy

**Keywords:** *Aelurostrongylus abstrusus*, Cat, Diagnosis, Italy, Metastrongyloidea, Molecular biology, *Troglostrongylus brevior*, *Troglostrongylus subcrenatus*

## Abstract

**Background:**

*Aelurostrongylus abstrusus* is currently regarded as the main metastrongyloid infesting domestic cats, whereas the reports of *Troglostrongylus* spp. in domestic and wild felids largely remain anecdotic. This paper reports on pulmonary infestation caused by *Troglostrongylus brevior* and *Troglostrongylus subcrenatus* in two kittens and describes, for the first time, associated clinical presentations and pathological features. Morphometrical, molecular and phylogenetic analyses have also been conducted to differentiate here the examined *Troglostrongylus* species from *A. abstrusus*, towards a clearer delineation of metastrongyloids affecting cats.

**Methods:**

Two kittens were referred for respiratory distress and hospitalized with a diagnosis of severe aelurostrongylosis, based on the presence of metastrongyloid larvae in the faeces. Despite prompt treatment, kittens died within 48 hours. Both kittens were submitted to necropsy to determine the cause of death.

**Results:**

At necropsy, nematode specimens were found in the trachea, bronchi and bronchioles and were associated with respiratory signs (i.e., dyspnoea, polypnea, severe coughing and nasal discharge). Morphology and measurements of adult parasites found allowed the unequivocal identification of *T. brevior* and *T. subcrenatus*, even if first stage larvae were rather similar to those of *A. abstrusus*. Briefly, *T. brevior* and *T. subcrenatus* larvae were shorter in length and lacking the typical knob-like terminal end of *A. abstrusus*. Molecular and phylogenetic analyses corroborated morphological identification and provided data on mitochondrial and ribosomal DNA genes of *T. brevior*.

**Conclusions:**

Data presented here indicate that *T. brevior* and *T. subcrenatus* may cause major respiratory distress in domestic cats. Consequently, these two species should be included, along with *A. abstrusus,* in the differential diagnosis of cat bronchopulmonary affections and treatment protocols need to be evaluated. Through research on the biology, epidemiology and control of *Troglostrongylus* spp. infestations in domestic cats are advisable to implement current knowledge on these neglected metastrongyloids.

## Background

Nematode infestations of the cardio-pulmonary system of dogs and cats have recently gained thescientific interest of researchers and practitioners, due to the clinical severity of the conditions they cause and to their spread throughout many Europeancountries [1-3]. Among them, *Aelurostrongylus abstrusus* (Strongylida: Angiostrongylidae) is commonly regarded as the only metastrongyloid of domestic cats [4,5]. Broncho-pulmonary infestations by *A. abstrusus* are widespread throughout most European countries with prevalence rates ranging from 1% to 24.4%, according to sampled populations and detection procedures (e.g., copromicroscopy or molecular detection) [2,6-12]. Actually, other metastrongyloids have been reported as causative agents of respiratory infestation in domestic cats. For instance, *Oslerus rostratus* (Strongylida: Filaroididae) has been recorded to infest the pulmonary system of cats with prevalence rates up to 24% in Spain [13,14]. Meanwhile, *Troglostrongylus subcrenatus* (Strongylida: Crenosomatidae) was retrieved once at the necropsy of a domestic cat from Blantyre (Nyasaland, Malawi) [15] and, larvae of a yet unclassified *Troglostrongylus* sp. have recently been identified in faeces of domestic cats from Ibiza (Spain), which exhibited respiratory disease [16].

The genus *Troglostrongylus* encompasses four nematode species (i.e., * T. brevior T. subcrenatus T. troglostrongylus* and * T. wilsoni*), which are commonly found in the trachea and bronchi of wild felids [17-21]. Albeit scantly studied, * Troglostrongylus * spp. display an indirect life cycle similar to that of * A. abstrusus *, with terrestrial molluscs serving as intermediate hosts and small mammals (e.g., mice) as paratenic ones [4,22,23]. Larvae of * T. brevior * may develop in several species of molluscs (i.e., * Helicella barbesiana  Helicella vestalis  Limax flavus  Monacha syriaca  Retinella nitellina * and * Theba pisana *), becoming infective from 8 to 40 days depending on the environmental temperature (i.e., between 22°-27°C and between 4°-8°C, respectively) [17]. Nonetheless, the scant scientific information available on the distribution and pathogenicity of * Troglostrongylus * sp. in domestic cats [16] might indicate that this infestation occurs sporadically or that it is misdiagnosed with the commonest *A. abstrusus*.

This paper reports on pulmonary infestation by *T. brevior* and *T. subcrenatus* in two kittens and describes, for the first time, associated clinical presentations and pathological features. Additionally, morphometric, molecular and phylogenetic analyses have also been conducted to differentiate the *Troglostrongylus* species here examined from *A. abstrusus* towards a clearer delineation of metastrongyloids affecting cats.

## Methods

### Case description

A 3-month-old female privately owned kitten (case 1), and a 3-month-old female stray kitten (case 2) were referred to the Faculty of Veterinary Medicine of Messina (Sicily region, Italy) on April 2010 and June 2011. Both animals exhibited respiratory signs such as dyspnoea, polypnea, severe coughing and nasal discharge (see Additional file 1 and Additional file 2). In addition, case 1 had developed inappetence and lethargy in the 48 h prior to referral while case 2 was dehydrated and in poor general condition at the moment of the clinical examination. Thoracic radiographs, performed only for case 2, showed diffuse increase of radiograph density and marked bronchial pattern in diaphragmatic lobes. Leucocytosis (i.e., 26.3 × 10^3^/μl in case 1, and 20.9 × 10^3^/μl in case 2) was diagnosed while biochemistry was within normal limits in both cases. *Toxocara cati* eggs were detected at the copromicroscopy using the flotation method in case 1 and of nematode larvae, resembling those of *A. abstrusus*, were retrieved by the Baermann technique in both cases. Kittens were both hospitalized with a diagnosis of severe aelurostrongylosis and antiparasitic drugs (imidacloprid 10 mg/kg/moxidectin 1 mg/kg spot-on in case 1, and fenbendazole 50 mg/kg PO once every 24 h in case 2) were administered. Both kittens also underwent systemic therapy with intravenous fluids, antibiotics (amoxicillin 20 mg/kg IV twice every 24 h and enrofloxacin 5 mg/kg IV once every 24 h) and placed in an oxygen chamber but they did not show any improvement with *exitus* within 24-48 h after hospitalization. Animals were not enrolled in any experimental trial. Kittens were hospitalized and keep according to Animal Welfare and Good Clinical Practice (VICH GL9-GCP, 2 "Good Clinical Practice", CVMP, June 2000) guidelines. In both cases necropsy was requested and authorized by the owners.

### Pathological findings and parasite identification

Carcasses of both kittens were subjected to necropsy. Parasitic specimens collected from lungs were washed in saline solution and stored in 70% ethanol (case 1) and in formalin (case 2). Worms were individually mounted on slides by the glycerol-ethanol method and microscopically observed. Microscopic images and measures were taken by using a digital image processing system (AxioVision rel. 4.8, Carl Zeiss, Germany). Parasites were sexed and identified at the species level by morphometrical and morphological keys [15,17,23]. Slide-mounted specimens of both sexes were stored in the parasite collection of the Faculty of Veterinary Medicine of Messina. In addition, gross anatomical findings, anatomical localization of parasites, and their morphometric features were also compared with those from *A. abstrusus* from a road-killed stray cat (E.B. unpublished observations).

### Molecular procedures and analyses

The molecular identification was performed by extracting genomic DNA from two parasite specimens of case 1 using a commercial kit (DNeasy Blood & Tissue Kit, Qiagen, GmbH, Hilden, Germany) in accordance with the manufacturer’s instructions. Unfortunately, all attempts to extract genomic DNA from nematodes retrieved from case 2 failed, probably because the specimens were kept in formalin.

A mitochondrial partial cytochrome *c* oxidase subunit 1 gene (p*cox*1, ~400 bp), 18S (~1700 bp) and internal transcribed spacer 2 (ITS2, ~630 bp) of ribosomal RNA gene were amplified. In particular, p*cox*1 was amplified by using the degenerated set of primers T*cox*F (5'-TGGARYTRTCTAARCCNGG-3') and T*cox*R (5'-GGAGGATAHACHGTYCAHC-3'), which was designed, using the criteria of Sharrocks [24], on the basis of the consensus sequences obtained by the multiple alignment of sequences of Metastrongyloidea available in GenBank^TM^ (Table 1). The 18S, ITS2 and flanking sequences of the 5.8S and 28S rRNA genes were amplified by two sets of primers (NC18SF1: 5'-AAAGATTAAGCCATGCA-3' and NC5BR: 5'- GCAGGTTCACCTACAGAT-3'; D: 5'-GAGTCGATGAAGAACGCAG-3' and B: 5'-GAATTCTGGTTAGTTTCTTTTCCT-3', respectively) [25,26]. Each reaction for p*cox*1 and 18S consisted of 4 μl genomic DNA and 46 μl of PCR mix containing 2.5 mM MgCl_2_, 10 mM Tris–HCl, pH 8.3 and 50 mM KCl, 250 μM of each dNTP, 50 pmol of each primer and 1.25 U of Ampli Taq Gold (Applied Biosystems). Approximately 100 ng of genomic DNA were added to each PCR and samples without DNA were included with each batch of sample tested. The p*cox*1 were amplified using the following conditions: 95°C for 10 min (first polymerase activation and denaturation); followed by 35 cycles of 95°C for 1 min (denaturation); 48°C for 1 min (annealing), 72°C for 1 min (extension); and a final extension at 72°C for 7 min. The PCR of 18S and ITS2 were carried out by 30–40 cycles of 94°C for 30 sec (denaturation), 72°C for 1 min and 45 sec (extension), and annealing temperatures of 57–58°C for 30–45 sec, respectively. Polymerase activation and denaturation and final extension were similar to those above. All amplicons were resolved in ethidium bromide-stained (2%) agarose (Gellyphor, Euroclone, Italy) gels and sized by comparison with markers in the Gene Ruler^TM^ 1 kb DNA Ladder (MBI Fermentas, Vilnius, Lithuania). Gels were photographed by a digital documentation system (Gel Doc 2000, BioRad, UK). Amplicons were purified using Ultrafree-DA columns (Amicon, Millipore; Bedford, USA) and then sequenced directly using the *Taq* DyeDeoxyTerminator Cycle Sequencing Kit (v.2, Applied Biosystems) in an automated sequencer (ABI-PRISM 377). 

**Table 1:** **GenBank**^** TM **^**accession numbers (AN) of the Metastrongyloidea used in the phylogenetic analyses for 18S rRNA and the internal transcribed spacer 2 (ITS2) genes**

**DNA region**	**Species**	**Host**	**Country**	**AN**
18S	*Aelurostrongylus abstrusus*	*Felis catus*	-	AJ920366
	*Angiostrongylus costaricensis*	*Sigmodon hispidus*	-	EF514913
	*Angiostrongylus costaricensis*	-	-	DQ116748
	*Angiostrongylus dujardini*	*Myodes glareolus*	-	EF514915
	*Angiostrongylus malaysiensis*	*Rattus tiomanicus*	-	EF514914
	*Angiostrongylus vasorum*	*Vulpes vulpes*	-	EF514916
	*Angiostrongylus vasorum*	*Vulpes vulpes*	-	AJ920365
	*Crenosoma mephitidis*	*Mephitis mephitis*	USA	AY295805
	*Crenosoma* sp.	*Ailurus fulgens*	-	GU475120
	*Crenosoma striatum*	*Erinaceus europaeus*	UK	GU214747
	*Crenosoma vulpis*	*Vulpes vulpes*	-	AJ920367
	*Didelphostrongylus hayesi*	*Didelphis virginiana*	USA	AY295806
	*Filaroides martis*	*Neovison vison* (=*Mustela vison*)	Canada	AY295807
	*Halocercus invaginatus*	*Phocoena phocoena*	USA	AY295808
	*Metastrongyloidea* sp.	*Ailurus fulgens*	-	GU475121
	*Metastrongylus elongatus*	*Sus scrofa*	-	AJ920363
	*Metastrongylus salmi*	*Sus scrofa*	USA	AY295809
	*Muellerius capillaris*	*Ovis aries*	USA	AY295810
	*Nippostrongylus brasiliensis*	*Rattus* sp.	-	AJ920356
	*Oslerus rostratus*	*Lynx rufus*	USA	GU946678
	*Oslerus osleri*	*Canis latrans*	USA	AY295812
	*Otostrongylus circumlitus*	*Mirounga angustirostris*	USA	AY295813
	*Otostrongylus* sp.	*-*	-	OSU81589
	*Parafilaroides decorus*	*Zalophus californianus*	USA	AY295814
	*Parafilaroides* sp.	-	-	U81590
	*Protostrongylus rufescens*	*Ovis aries*	-	AJ920364
	*Pseudalius inflexus*	*Phocoena phocoena*	USA	AY295816
	*Skrjabingylus chitwoodorum*	*Mephitis mephitis*	USA	AY295819
	*Stenurus minor*	*Phocoena phocoena*	USA	AY295817
	*Torynurus convolutus*	*Phocoena phocoena*	USA	AY295818
	*Troglostrongylus* sp.	*Felis catus*	Spain	GU946677
	*Troglostrongylus wilsoni*	*Lynx rufus*	USA	AY295820
ITS2	*Aelurostrongylus abstrusus*	*Felis silvestris catus*	Italy	DQ372965
	*Aelurostrongylus abstrusus*	*Felis silvestris catus*	Italy	EU034168
	*Angiostrongylus dujardini*	*Apodemus sylvaticus*	France	GQ181113
	*Angiostrongylus vasorum*	*Canis familiaris*	Germany	GU045375
	*Elaphostrongylus alces*	*Alces alces*	Sweden	AF504028
	*Elaphostrongylus alces*	*Alces alces*	Sweden	AF504034
	*Elaphostrongylus cervi*	*Cervus elaphus*	New Zealand	AF504032
	*Elaphostrongylus cervi*	*Cervus elaphus*	New Zealand	AF504026
	*Elaphostrongylus rangiferi*	*Rangifer tarandus*	Canada	AY648408
	*Elaphostrongylus rangiferi*	*Rangifer tarandus*	Canada	AF504027
	*Elaphostrongylus rangiferi*	*Rangifer tarandus*	Canada	AF504033
	*Halocercus invaginatus*	*-*	-	FJ787301
	*Metastrongylus asymmetricus*	*Sus scrofa*	-	Y08006
	*Metastrongylus elongatus*	*Sus scrofa*	Estonia	AJ305377
	*Metastrongylus elongatus*	*Sus scrofa*	Estonia	AJ305376
	*Metastrongylus elongatus*	*Sus scrofa*	Estonia	AJ305381
	*Metastrongylus pudendotectus*	*Sus scrofa*	-	Y08008
	*Muellerius capillaris*	*Ovis aries*	Canada	AY679527
	*Muellerius capillaris*	*Ovis aries*	Canada	AY679528
	*Nematodirus battus*	*-*	-	JF345079
	*Otostrongylus circumlitus*	*Mirounga angustirostris*	-	AF130455
	*Parelaphostrongylus odocoilei*	*Ovis aries*	Canada	AY648402
	*Parelaphostrongylus odocoilei*	*Odocoileus hemionus hemionus*	Canada	AF504031
	*Parelaphostrongylus tenuis*	*Odocoileus virginianus*	Canada	AF504035
	*Parelaphostrongylus tenuis*	*Odocoileus virginianus*	Canada	AF504029
	*Pseudalius inflexus*	*-*	-	FJ767935
	*Stenurus minor*	*-*	-	FJ787302
	*Torynurus convolutus*	*-*	-	AY464532

*Nippostrongylus brasiliensis* and *Nematodirus battus* were used as outgroups.

Sequences were determined from both strands (using the same primers individually as for the PCR) and the electropherograms verified by eye. In order to ensure open reading frames, the nucleotide sequence of p*cox*1 was conceptually translated into amino acid sequence using the invertebrate mitochondrial code MEGA5 software [27]. Sequences for all genes were compared with those available in the GenBank™ database by Basic Local Alignment Search Tool (BLAST) [28]. In order to investigate the relationships among metastrongyloids affecting wild and domestic carnivores, sequences of 18S and ITS2 were analysed with those available in GenBank^TM^ (Table 1) and the evolutionary history was inferred using the neighbour-joining method [29]. The bootstrap consensus trees inferred from over 7,000 replicates were taken to represent the evolutionary history of the taxa analysed [30]. Branches corresponding to partitions reproduced in less than 50% bootstrap were collapsed and the percentage of replicate trees in which the associated taxa clustered together in the bootstrap test was shown next to the branches [30]. Phylogenetic analyses were conducted in MEGA5 software [27]. Sequences of *Nippostrongylus brasiliensis *(Strongylida: Heligmonellidae) and *Nematodirus battus* (Strongylida: Molineidae) were used as outgroups. The nucleotide sequences analyzed in this paper are available in the GenBank^TM^ database under accession numbers: JX290562 (18S); JX290563 (*cox*1); JX290564 (ITS2).

## Results

### Pathological findings

**Figure 1:**
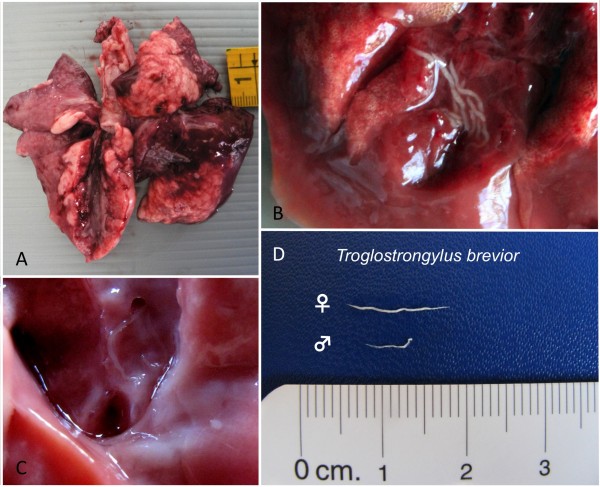
**Gross anatomy and parasites collected at the necropsy of case 1.** Lungs were congested and swollen, lobular bronchopneumonia was noticed in lobes of the right lung (**A**). Several parasites were present in respiratory tracts (**B-C**). A total of twelve slender worms (up to 13 mm length), identified as *Troglostrongylus brevior* (**D**), were collected from bronchi and bronchioles.

In case 1, lungs were congested and swollen with multifocal areas of haemorrhages, diffused hepatisation and lobular bronchopneumonia in lobes of the right lung (Figure 1A). The cut surface was oedematous with catarrhal exudate draining from small airways and parasites were present in respiratory tracts (Figure 1B-1C). Organs of abdominal cavity appeared normal.

**Figure 2:**
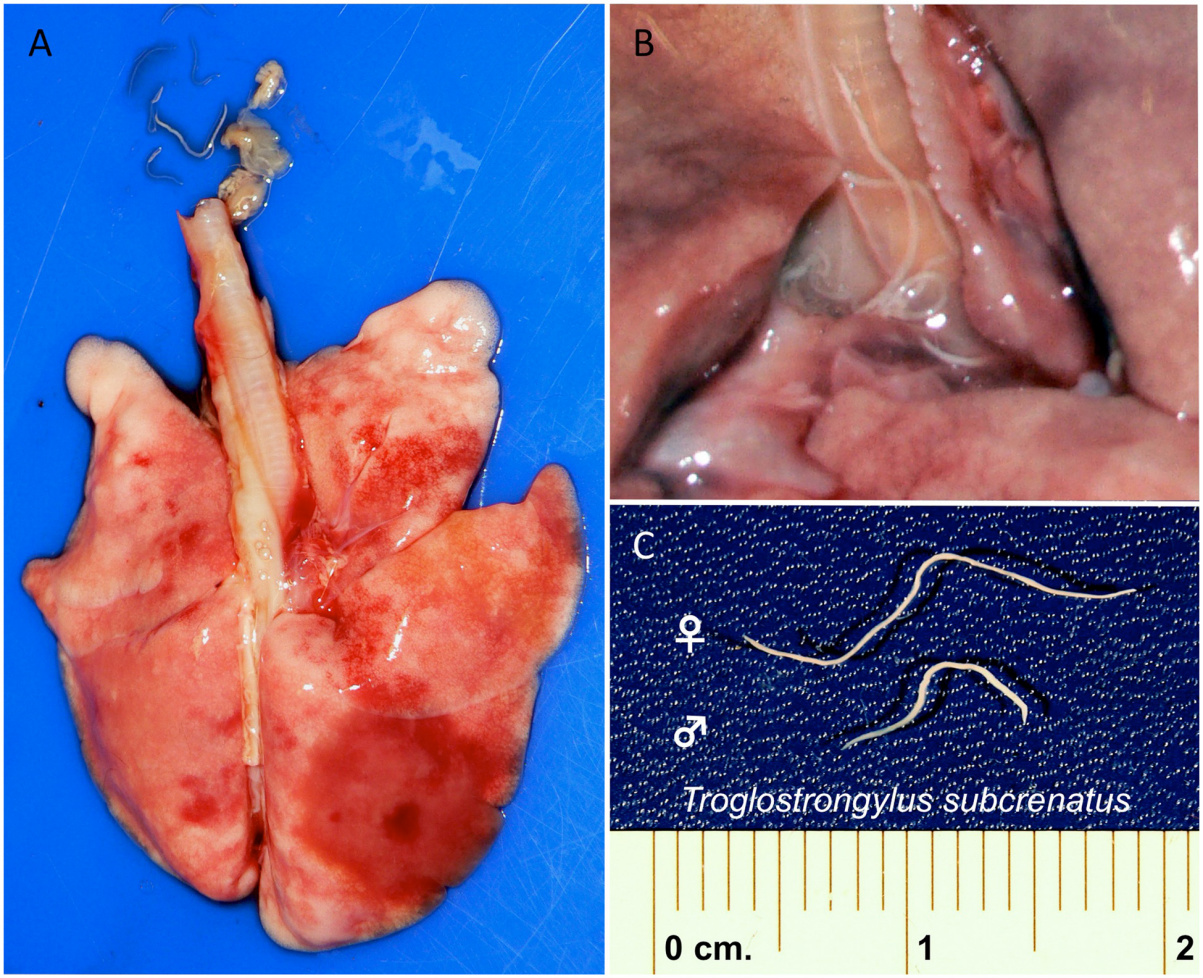
**Gross anatomy and parasites collected at the necropsy of case 2.** Lungs were diffusely congested and swollen with a large area of consolidation in the right diaphragmatic lobe (**A**). Parasites mixed with catarrhal exudate were revealed when cutting the trachea (**B**), A total of twenty-two whitish nematodes (up to 24 mm length), identified as *Troglostrongylus subcrenatus* (**C**) were collected from the trachea and large bronchi.

In case 2, lungs were diffusely congested and swollen with a large area of consolidation in the right diaphragmatic lobe (Figure 2A). Nematodes mixed with catarrhal exudate were revealed when the trachea was cut (Figure 2B). Other organs and apparatus were normal at gross examination. In both cases, the respiratory failure was identified as the cause of death.

Slender and whitish nematodes (12 from case 1 and 22 from case 2) were collected from bronchi, bronchioles (Figure 1D) and from trachea and large bronchi (Figure 2C) in case 1 and 2, respectively.

### Parasite identification

**Figure 3:**
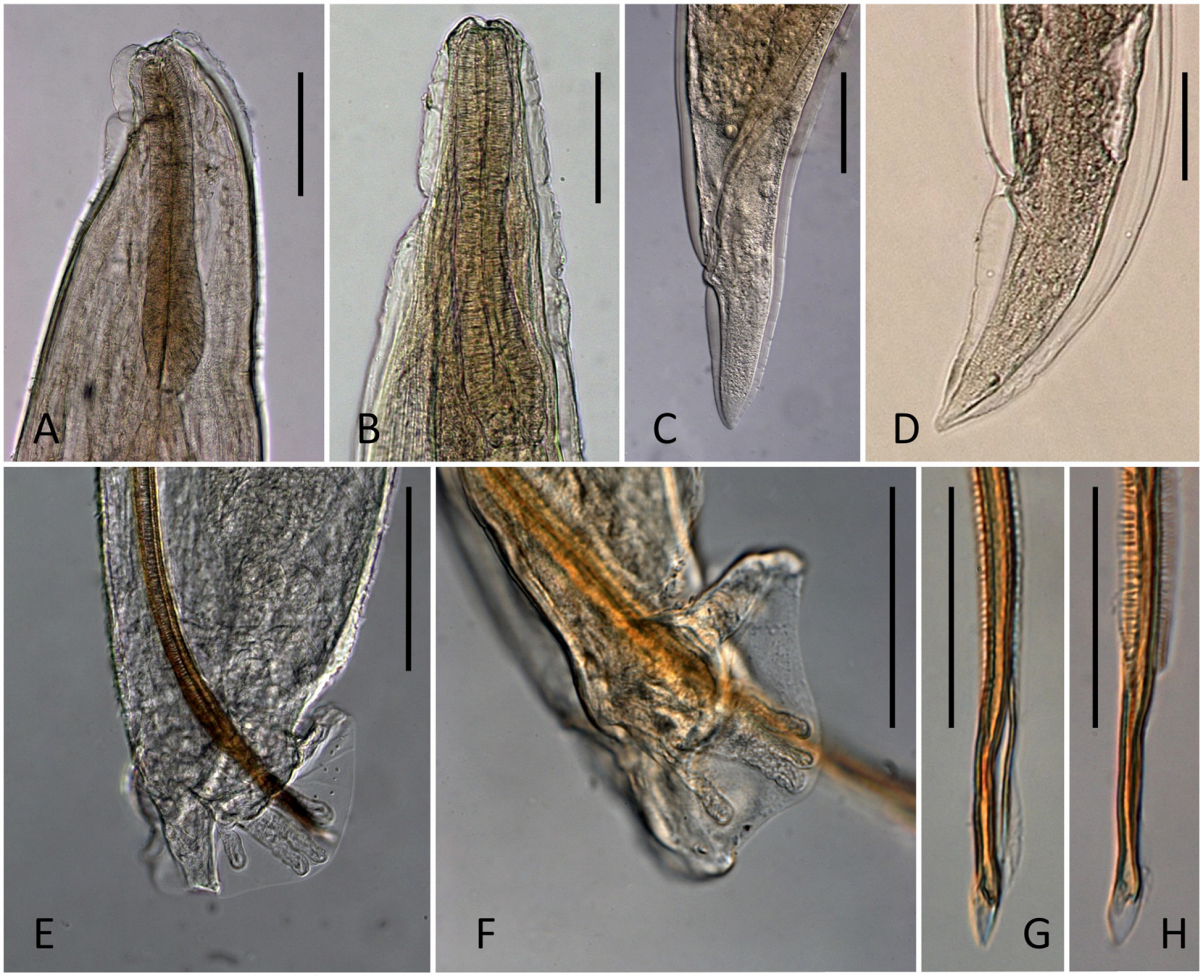
**Light microscope images depicting morphometrical differences of cephalic region, caudal region, male bursa and spicules between *Troglostrongylus brevior *(A, C, E and G) and *Troglostrongylus subcrenatus *(B, D, F and H).** Scale bars = 100 μm (A-F), 50 μm (G-H).

**Figure 4:**
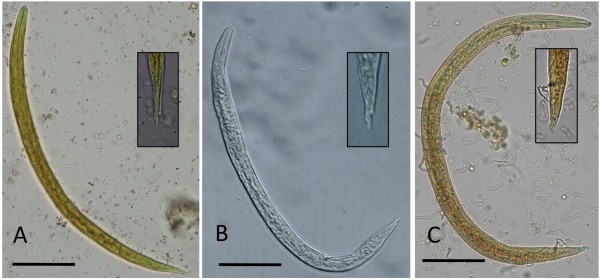
**Light microscope images of first stage larvae (L1) of *Troglostrongylus brevior *(A), *Troglostrongylus subcrenatus *(B) and *Aelurostrongylus abstrusus *(C) Magnification of caudal region is provided for each species.** Scale bar = 50 μm.

Parasites collected from case 1 were thin and cylindroid presenting a cuticle that was inflated and thrown into folds. The oesophagus was short and club-shaped, the stoma small and weakly developed. The excretory gland was large and extending almost to the posterior extremity of the body opening, in both sexes, at the first third of the oesophagus (Figure 3A). Nine females and three males were identified. Briefly, females were 6–13 mm in length and 0.34–0.43 mm in width with a conical and short tail (125–168 μm) and inflated cuticle (Figure 3C; Table 2). The gravid uterus contained larvated eggs. Males were 5–6 mm in length and 0.43 mm in width and the oesophagus was 281–301 μm length (Table 2). The caudal edge of males showed a well-developed, undivided bursa composed by a dorsal ray elongated with four apical papillae, externo-dorsal and antero-lateral rays were well separated, postero- and medio-lateral rays joined except for the distal third, ventral rays were short and fused except at their end (Figure 3E). The spicules (0.60–0.63 mm) were equal in length, thin and transversely striated for most of their extent with serrated edges (Figure 3G; Table 2). First stage larvae (L1) (339.3 μm in length and 18.6 μm in width) had a rhabditoid oesophagus, numerous intestinal cells filled with granules and pointed tail with pronounced dorsal cuticular spine and a shallower ventral one (Figure 4A; Table 3). Morphology and measurements of adult and larval nematodes collected from case 1 were consistent with those of *T. brevior*[17]. 

**Table 2:** **Anatomical localization and morphometrical features of adult stages of *Aelurostrongylus abstrusus*, *Troglostrongylus brevior *and *Troglostrongylus subcrenatus***

**Species**	**Localization**	**Sex**	**Length (mm)**	**Width**	**Oesophagus**	**Tail (anus-caudal end)**	**Spicule length**
*Aelurostrongylus abstrusus* (Gerichter, 1949)	Pulmonary parenchyma	Male	5–6	54–64	240–270	-	130–220
		Female	10–10.4	80	300	27–29	-
*Troglostrongylus brevior*							
(present study)	Bronchi and bronchioles	Male (n = 3)	5–6	294–365	281–301	-	600–631
		Female (n = 6)	6–13	335–430	272–298	125–168	-
*Troglostrongylus subcrenatus*							
(present study)	Trachea and bronchi	Male (n = 7)	9–10	285–305	255–302	-	613–686
		Female (n = 15)	20–24	486–542	283–312	178–189	-

Measurements are expressed in microns, otherwise specified.

**Table 3:** **Mean measurements (microns) of first stage larvae (L1) of *Aelurostrongylus abstrusus *, *Troglostrongylus brevior *and *Troglostrongylus subcrenatus***

**Species (number of specimens)**	**First stage larvae (L1)**	
	**Mean length**	**Mean width**
*Aelurostrongylus abstrusus* (n = 40)	399.1 ( ± 11.3)	18.5 ( ± 1.2)
*Troglostrongylus brevior* (n = 40)	339.3 ( ± 14.1)	18.6 ( ± 1)
*Troglostrongylus subcrenatus* (n = 10)	280.7 ( ± 17.9)	15.5 ( ± 1.7)

Parasites collected from case 2 were stout and filiform nematodes of medium size with a cuticle conspicuously inflated at the anterior end. The oesophagus was club-shaped. The excretory pore opening coincided with the ventral cuticular groove at about the middle of the oesophagus length (Figure 3B). A total of 15 females and seven males were identified. Females (20–24 mm in length and 486–542 μm in width) presented a conical and bluntly pointed tail (Figure 3D; Table 2) with the vulva opening posterior to the middle of body and the anus at 178–189 μm from the caudal end (Figure 3D). The uteri were filled with larvated eggs. Males (9–10 mm in length and 285–305 μm in width) presented an undivided bursa composed of a single dorsal stout ray, bearing papillae at the tip and externo-dorsal rays originating independently. Postero- and medio-lateral rays were fused over half or more of the length, antero-lateral rays were single and ventral rays fused for almost their extension (Figure 3F). The spicules (613–686 μm in length) were equal in size and shape, slender and finely striated except for their distal edges. Each spicule was split into two spikes (Figure 3H). First stage larvae (280.7 μm in length and 15.5 μm in width) had a rhabditoid oesophagus and pointed tail bearing typical dorsal and ventral cuticular spines (Figure 4B; Table 3). Morphology and measurements of adult and larval nematodes collected from case 2 were all consistent with those of *T. subcrenatus*[15,31]*.*

Morphological and morphometrical features of adult parasites collected from cases 1 and 2 are reported in Table 2 and compared to those of *A. abstrusus*. In spite of the differences in anatomical localization and body size of adult parasites, first stage larvae of *A. abstrusus*, *T. brevior* and *T. subcrenatus* are rather similar, except for their slightly differing body lengths and the knob-like terminal end of *A. abstrusus* (see Figure 4 and Table 3).

**Figure 5:**
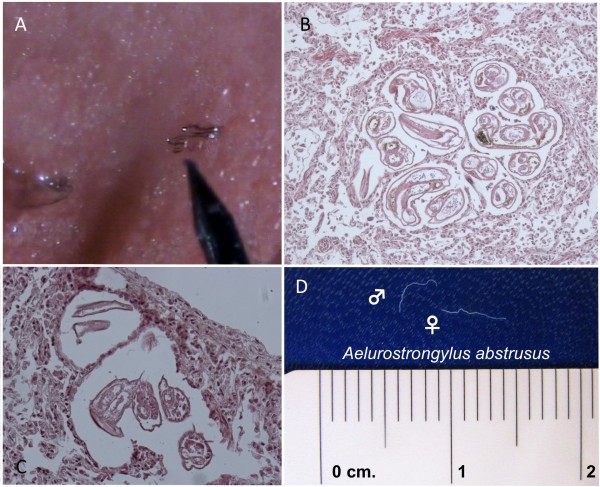
**Sub-pleural nodules caused by *Aelurostrongylus abstrusus *in the lungs of a cat (A) Histological sections (HE) showing localization of adult worms of *A. abstrusus *in the lung parenchyma (B) and in sub-pleural nodules (C) Adult worms (male and female) of *A. abstrusus *(D).**

Adult parasites of *A. abstrusus* were found in respiratory bronchioles and lung parenchyma and gross lesions were characterized by the presence of small greyish subpleural nodules (Figure 5A) containing a mass of larvae, eggs and adult parasites (Figure 5B). Alveoli containing parasites appeared dilated with disrupted septa and encircled by eosinophilic, neutrophilis and mononuclear infiltrate (Figure 5C).

PCR amplification of each target region characterized for nematodes from case 1 resulted in amplicons ranging from ~400 bp (p*cox*1) to ~1700 bp (18S) and ~630 bp (ITS2). The BLAST analysis of p*cox*1 gene sequences showed the closest homology (91%) with that of *Necator americanus* (GenBank accession number: AF303151) whereas ITS2 revealed a high homology (99%, fragment of 89 bp) when compared with *Angiostrongylus cantonensis* and *Angiostrongylus vasorum* (Strongylida: Angiostrongylidae) (GenBank accession numbers: HQ540551 and GU045376, respectively). The 18S sequence here produced was identical (homology of 100%) to *Troglostrongylus* sp. (GenBank accession number: GU946677).

**Figure 6:**
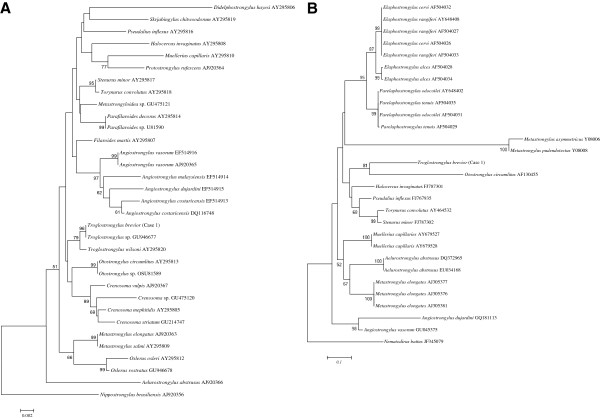
**Phylogenetic trees based on regions of 18S (A) and ITS2 (B) ribosomal DNA sequence data, compared with those of metastrongyloids available in GenBank**^**TM**^**.** The trees were constructed using neighbour-joining (NJ) method (7000 replicates).

The phylogenetic analyses of the sequence here produced for 18S and ITS2 genes showed a close evolutionary relationship among worms of case 1 with those of *Troglostrongylus* spp., *Otostrongylus* spp. and *Crenosoma* spp. (Strongylida: Crenosomatidae) with relatively strong nodal support, to the exclusion of other metastrongyloids (Figure 6A-B). In particular, the 18S tree revealed that the nematode was close to a *Troglostrongylus* sp. from Spain (Figure 6A).

## Discussion

This paper is the first of all reports on clinical signs and gross lesions associated with *T. brevior* and *T. subcrenatus* infestation in two domestic kittens. The delineation between these two species of metastrongyloids and the most known *A. abstrusus* is pivotal for the aetiological diagnosis of helminthic broncho-pulmonary disease of cats. These two troglostrongylids have been neglected for a long time or underestimated and deserve further investigations to assess their actual geographical distribution and clinical significance, especially in young cats. Indeed, *Troglostrongylus* spp. have been reported as causative agents of respiratory conditions in many species of wild felids (e.g., *Lynx rufus, Panthera pardusPanthera tigris*) [17-21], but their occurrence in domestic cats was considered negligible [22]. Recently, a *Troglostrongylus* sp. detected in domestic cats exhibiting respiratory signs renewed the scientific interest on this genus [16]. The 18S sequences herein produced for *T. brevior* and those derived from the parasitic specimens recently identified [16] provided an *a posteriori* aetiological identification for the causative agents of the cases above from Spain. This evidence might suggest that *Troglostrongylus* spp. are more diffuse than currently estimated and that the limited number of records of *T. brevior* and *T. subcrenatus* infesting domestic cats could be due to the close morphological features shared among their L1s (i.e., the parasitic stage mostly used for the diagnosis) with those of *A. abstrusus*. On the other hand, it cannot be ruled out that troglostrongylosis in domestic cats is limited to areas where cats are more frequently exposed to intermediate and/or paratenic hosts. Furthermore, molluscs of genera (i.e., *Helicella* spp. *Limax* spp. and *Theba* spp.) are common in the locality where both cats reported in this paper came from [32].

Accordingly, almost all data available on lungworms affecting cats are based on copromicroscopical findings [6-12], which might lead to a misdiagnosis unless a thorough morphological and morphometric analysis is undertaken. Even if not easily achievable, an accurate evaluation of morphometrical features (i.e., total length and tail morphology) of L1s may lead to a discrimination among *T. breviorT. subcrenatus* and *A. abstrusus*, being larvae of *Troglostrongylus* spp. shorter in length and devoid of the typical knob-like terminal end (see Figure 4 and Table 3).

Molecular and phylogenetic analyses presented here provide, for the first time, data on mitochondrial and ribosomal gene sequences of *T. brevior*, giving a dataset for its identification. Based on the analysis of the 18S gene, the aetiological cause of infestation in cats from Ibiza was *T. brevior*[16]. The phylogenetic analysis of 18S further confirms the molecular analysis, clustering *T. brevior* within other *Troglostrongylus* spp. infecting domestic carnivores. Nonetheless, further studies should be undertaken to differentiate *T. brevior* and *T. subcrenatus* molecularly in order to better understand their pathogenicity, biology and bionomics (including the role of different species of molluscs as intermediate hosts) and infer their potential spread throughout Europe. A molecular characterization of these two species might contribute to assessing a specific and sensitive molecular tool for the detection of both species in faeces and pharyngeal swabs, as recently validated for *A. abstrusus*[33].

The greater body size of *T. brevior* and *T. subcrenatus* in comparison with *A. abstrusus* and their anatomical localisation in the upper respiratory airways (i.e., bronchioles and large bronchi) also reflects differences in the presentations and severity of clinical signs they cause in cats. Indeed, both clinical cases here described exhibited severe respiratory distress and had a lethal outcome due to respiratory failure. Accordingly, most of the lung damage observed at the gross examination was associated with the presence and action of nematode specimens. This clinical picture differs from that caused by *A. abstrusus*, which generally induces minor signs and is reported as an often self-limiting parasite [1,5,34]. Nonetheless, the clinical presentation caused by *Troglostrongylus* spp. and *A. abstrusus* may overlap in simultaneously infested animals, thus making any definitive aetiological diagnosis based on respiratory signs difficult.

*Troglostrongylus brevior* and *T. subcrenatus* share similar biology with *A. abstrusus*, all involving mollusc intermediate hosts in their life cycle [4,17,22] and thus, most likely, occupying the same ecological niches and occurring simultaneously in a given host population, as confirmed by co-infestation in the same cat population from Ibiza [16]. Due to their capacity of developing to the infective stage under different climatic conditions in many species of intermediate hosts, troglostrongylids may have a potential broader distribution than currently believed. Indeed, larvae of *T. brevior* show the highest resistance in the environment, developing to the infective stage at low temperatures (i.e., 4-8°C in 40 days) but not *A. abstrusus*, even when kept up to 7 months at the same conditions [17]. Under optimal conditions (i.e., 22-27°C) *T. brevior* displays the shortest development time (i.e., 8 days) known for any metastrongyloid species [17], suggesting that this species may potentially express a higher parasitic pressure for feline populations than *A. abstrusus*.

## Conclusion

Data presented here indicate that *T. brevior* and *T. subcrenatus* may occur in domestic cats causing overt respiratory disease. Moreover, these two species should be included, along with *A. abstrusus,* in the differential diagnosis of bronchopulmonary affections in cats. Although the delineation of L1s among metastrongyloids is achievable using morphometrical criteria, molecular biology tools should be considered and implemented toward enhancing current knowledge on the biology, epidemiology and control of *Troglostrongylus* spp. infestations in domestic cats.

## Competing interest

The authors declare that they have no competing interests.

## Authors’ contributions

EB conceived the study, documented clinical cases, described parasites and drafted the manuscript. GG and SG collected parasites, performed the morphological identification and revised the drafts of the manuscript. GA and MSL carried out the molecular genetic studies, sequence alignment and phylogenetic analyses, and drafted the manuscript. DT and FDT revised the drafts of manuscript. DO participated in molecular genetic and phylogenetic studies and revised and implemented the drafts of manuscript. All authors read and approved the final version of manuscript.

## Supplementary Material

Additional file 1 **Dyspnoea caused by *Troglostrongylus brevior *Movie shows severe inspiratory and expiratory dyspnoea associated with *Troglostrongylus brevior *infestation.** Click here for file

Additional file 2 **Respiratory symptoms caused by *Troglostrongylus brevior *Movie shows respiratory symptoms (coughing and dyspnoea) observed in a kitten infested by * Troglostrongylus brevior.*** Click here for file
